# Predicting nickel concentration in soil using fractional-order derivative and visible-near-infrared spectroscopy indices

**DOI:** 10.1371/journal.pone.0302420

**Published:** 2024-08-01

**Authors:** Jianfei Cao, Wei Liu, Yongyu Feng, Jianhua Liu, Yuanlong Ni

**Affiliations:** 1 College of Geography and Environment, Shandong Normal University, Jinan, China; 2 Shandong Yuanhong Survey Planing and Design CO.,LTD, Jinan, China; 3 Shandong Provincial Institute Land Spatial Data and Remote Sensing Technology, Jinan, China; 4 Jinan Institute of Surveying and Mapping, Jinan, China; ICAR Central Coastal Agricultural Research Institute, INDIA

## Abstract

Accurate monitoring and estimation of heavy metal concentrations is an important process in the prevention and treatment of soil pollution. However, the weak correlation between spectra and heavy metals in soil makes it difficult to use spectroscopy in predicting areas with a risk of heavy metal pollution. In this paper, a method for detection of Ni in soil in eastern China using the fractional-order derivative (FOD) and spectral indices was proposed. The visible-near-infrared (Vis-NIR) spectra were preprocessed using the FOD (range: 0 to 2, interval: 0.1) to solve the problems of baseline drift and overlapping peaks in the original spectra. The product index (PI), ratio index (RI), sum index (SI), difference index (DI), normalized difference index (NDI), and brightness index (BI) were applied and compared. The results showed that the spectral detail increased as the FOD increased, and the interference of the baseline drift and overlapping peaks was eliminated as the spectral reflectance decreased. Furthermore, the FOD extracted the spectral sensitivity information more effectively and improved the correlation between the Vis-NIR spectra and the Ni concentration, and the NDI had a maximum correlation coefficient (r) of 0.803 for order 1.9. The estimation model based on the NDI dataset constructed after FOD processing had the best performance, with a validation accuracy $R_{P}^{2}$ of 0.735, RMSE of 3.848, and RPD of 2.423. In addition, this method is easy to carry out and suitable for estimating other heavy metal elements in soil.

## 1. Introduction

Heavy metal pollution is an important part of soil contaminant control. Heavy metals in soil can cause losses in agriculture and animal husbandry, and can ultimately harm the human body through the ecosystem cycle [1–3]. Despite the large amount of attention that has been paid to heavy metal (arsenic, cadmium) pollution due to their toxic properties, few studies have been conducted on some heavy metals such as nickel [4]. According to previous research, the nickel in soil mainly comes from the burning of fossil fuels, treatment of sewage, application of phosphate fertilizer, and mining activities [5, 6]. The nickel in soil is highly toxic and was listed as a class 1 carcinogen by the World health Organization (WHO) in 2017.

The monitoring of heavy metals in soil is difficult and vital due to the characteristics of heavy metal pollution: low contents, strong pollution, difficult to detect, and irreversible [7, 8]. However, conventional chemical analysis methods for heavy metals in soil are complex and expensive, have a long detection cycle, and easily cause secondary damage to the soil environment. Thus, they cannot meet the requirements of rapid and continuous dynamic monitoring of metals in soil at present [9]. Recently, visible and near-infrared (Vis-NIR) spectroscopy has been applied in the monitoring of soil properties due to its advantages of a high resolution, strong spatial continuity, lower cost, and easy acquisition [10–12].

Several studies have preprocessed Vis-NIR data using logarithmic transformation, reciprocal transformation, and first and second-order derivative transformation in combination with random forest (RF), support vector machine (SVM), stepwise regression, and other algorithms to build quantitative estimation models of heavy metal contamination of soils [13–16]. Among these methods, Vis-NIR spectroscopy has good feasibility for the rapid estimation and quantitative inversion of heavy metal contaminants. However, the contents of heavy metals in soil are very small, and their signals are weak. In these studies, the pretreatment of the spectral data was limited to the first and second-order derivatives and simple logarithm and reciprocal variations, which ignored the details of the spectral gradient process. Therefore, the effect of this method was better in areas with high metal concentrations, but it was less effective in areas with light pollution. In addition, the spectral signal is interfered with by soil salinity, the diameter of the soil particles, and other factors [17, 18], so there is a great deal of noise in the spectral data, which affects the emission of the information about the effective sensitivity band. Therefore, it is difficult to construct an efficient and accurate estimation model of heavy metal contamination of soil using this method.

For estimation models, the target information must be strong enough to ensure that the model can detect the numerical relationship between the variables. The fractional-order derivative (FOD) is a distinctive method that extends the traditional integer-order derivative technique to any order. It can make full use of the entire spectrum and mine the details of the spectral transformation [19–21], and it is characterized by integrity and memorability. To date, the FOD has been applied in signal and image processing, digital filtering, and other fields [22]. Recently, the FOD has also been introduced into Vis-NIR spectroscopy. Some scholars have demonstrated the feasibility of using the FOD in hyperspectral research. After pretreatment of the spectral data using the partial derivative, quantitative estimations of the soil’s salinity, organic carbon content, and plant biomass were made [23–25].

There is a great deal of spectral features and noise in the FOD pretreatment process, which is harmful to the extraction of the sensitivity data correlated to Ni in soil. The spectral index is an effective tool that can help extract the sensitivity data, and it has a wide range of applications. Several studies have used the spectral index for the quantitative inversion of the chlorophyll content and soil salinity and have found that the two-dimensional spectral index is better than the one-dimensional single band [26, 27]. Several scholars had achieved certain results when combining the spectral index with remote sensing images to study large-scale deforestation and environmental degradation [28, 29]. The spectral index has used in the field of remote sensing, and it can detect the nonlinear relationship between variables and effectively reduce the impact of the noise in spectral data. However, few studies have been conducted on the monitoring of the concentration of the heavy metal Ni using the spectral index and FOD.

In this study, soil samples and Vis-NIR spectral data were used to construct an accurate detection model of the nickel concentration in a low heavy metal pollution area in eastern China. The research main objectives were as follows: (1) to analyze the effect of FOD pretreatment on the reflectance, (2) to investigate the correlation between Ni concentration and Vis-NIR spectroscopy indices processed by different FOD transformations and construct the band combination datasets, and (3) to evaluate and compare the accuracy of estimating Ni concentration based on band combination dataset extracted from different spectral indices.

## 2. Materials and methods

### 2.1 Study area

**Fig 1:**
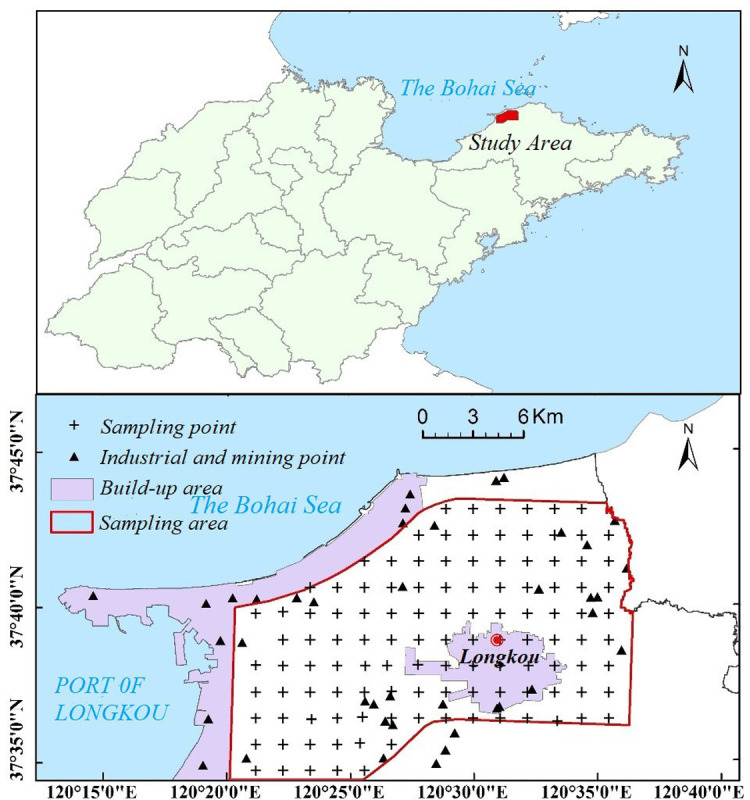
Maps of the study area (the figure above shows the location of the region, and the figure below shows the layout of sampling points).

The research area is located in Longkou City, Yantai City, Shandong Province, eastern China, in the northern part of the Jiaodong low hills (Fig 1). It has a typical temperate monsoon climate with rainy summers and dry winters. The local soils mainly include brown soil, cinnamon soil, and marine soil, among which cinnamon soil is the most widely distributed soil type and is mostly distributed in the hilly areas. The brown soils are mainly distributed in the hills and mountains, while the marine soil is distributed along the rivers and coastal beaches. The study area has fertile soil and is an important agricultural production area. The main crops are winter wheat and corn. In addition, the study area includes large industrial areas, residential areas, and coal mining bases. Industrial activities, transportation, coal mining, and other activities produce a great deal of sewage and waste gas. Longkou City has become an area rich in common heavy metals due to the emissions of waste gas and sewage. The heavy metals in the exhaust gas accumulate in the atmosphere and enter the soil. Sewage treated in sewage treatment plants is discharged into the rivers. Local agricultural irrigation water is diverted from rivers containing treated sewage, further contributing to the increase in the spread of heavy metals in the area. Therefore, high-precision monitoring of the heavy metals in the soil is very important for the development of land resource management and agricultural protection in Longkou City.

### 2.2 Sample collection and Ni analysis

In November 2018, 126 soil samples were collected in Longkou City. The five-point sampling method was adopted at each sampling location. The 0–10 cm surface soil interval was collected at each sample site and the sample plot extended 20 cm in the four cardinal directions. Impurities such as grass and branches were removed. The soil was stored in sealed bags, and the coordinates of each sampling point were recorded using the Global Positioning System (GPS). Each sample was about 1 kg. Previous studies have shown that the size of the soil particles can affect the attachment of heavy metals [30, 31]. The smaller the soil particle size, the stronger the heavy metal attachment. The samples were dried naturally in a dark room and were ground to 100 mesh to avoid the local ratio of heavy metal samples due to differences in soil particle diameter and grain size. Then, each sample was divided into two parts: one for analysis of the Ni content, and the other for acquisition of the Vis-NIR spectral data of the soil. The Ni content was determined using the atomic absorption method [6, 15]. Later the outliers were removed, and the remaining 108 soil samples were divided into two parts using the Kennard-Stone (K-S) method: 76 modeling sets (70%) and 32 validation sets (30%), which were later used to correct and test the accuracy and precision of the model.

### 2.3 Spectral measurements and preprocessing

**Fig 2:**
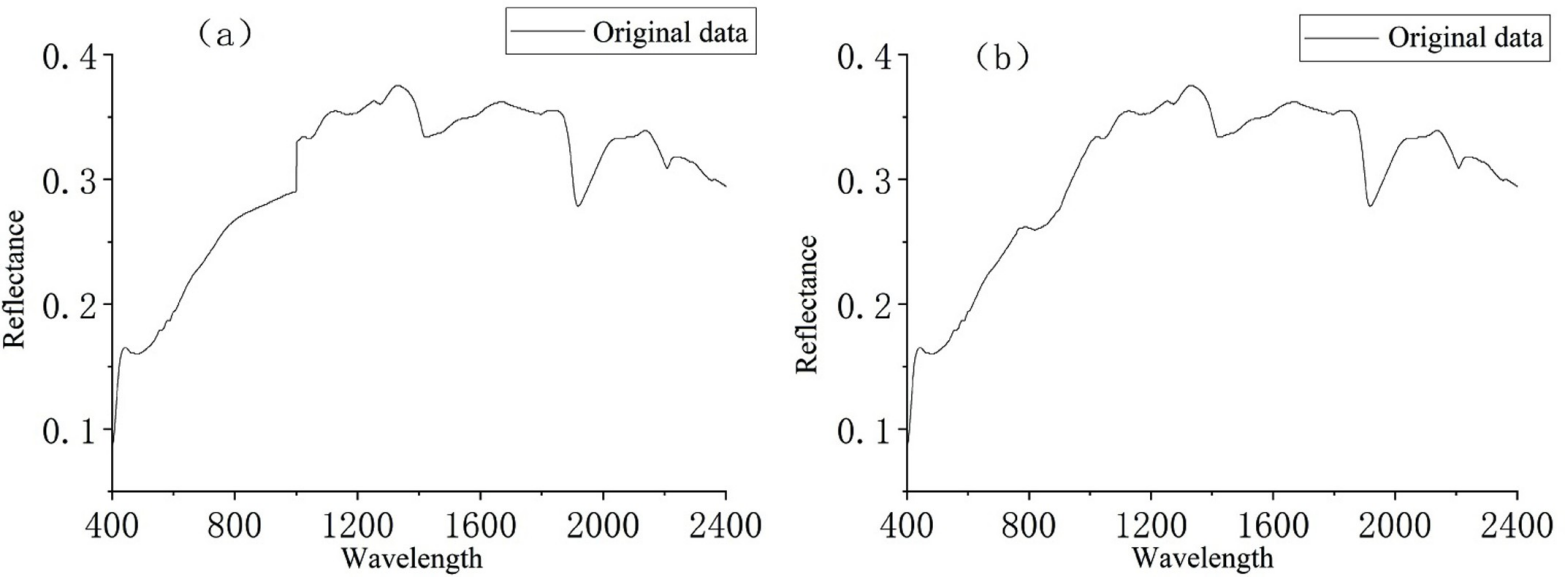
The original mean spectrum(a) and the mean spectrum of the removal of the jump point(b) by the Savitzky-Golay filtering tool.

The spectral information for 108 soil samples was collected using a controlled light source (two 50 W halogen lamps) and an ASD FieldSpec3 spectrometer in a dark room. The spectrum ranged from 350 nm to 2500 nm, and the sampling interval was 1.4 nm in the range of 350 nm to 1000 nm and 2 nm in the range of 1000 nm to 2500 nm. During the collection of the spectral data, two halogen lamps were placed 30 cm above the soil samples at an incidence angle of 45°. The spectrometer was corrected using whiteboard calibration before the spectral acquisition and after 10 tests of each soil sample. Ten spectra were measured for each soil sample, and the final average spectrum was used as the spectral curve of each sample after the abnormal spectra were removed. The data at 350–400 nm and 2401–2500 nm with a low signal-to-noise ratio (SNR) were removed. Because the ASD spectrum acquisition system consists of visible and near-infrared components, there is a jump point at 1000 nm, which affects the subsequent processing of the spectral data and the construction and extraction of the optimal spectral index. Therefore, the obtained spectral data were smoothed and the jump point correction was carried out using the Savitzky-Golay filtering tool in the ENVI software (Fig 2).

### 2.4 Methods

**Fig 3:**
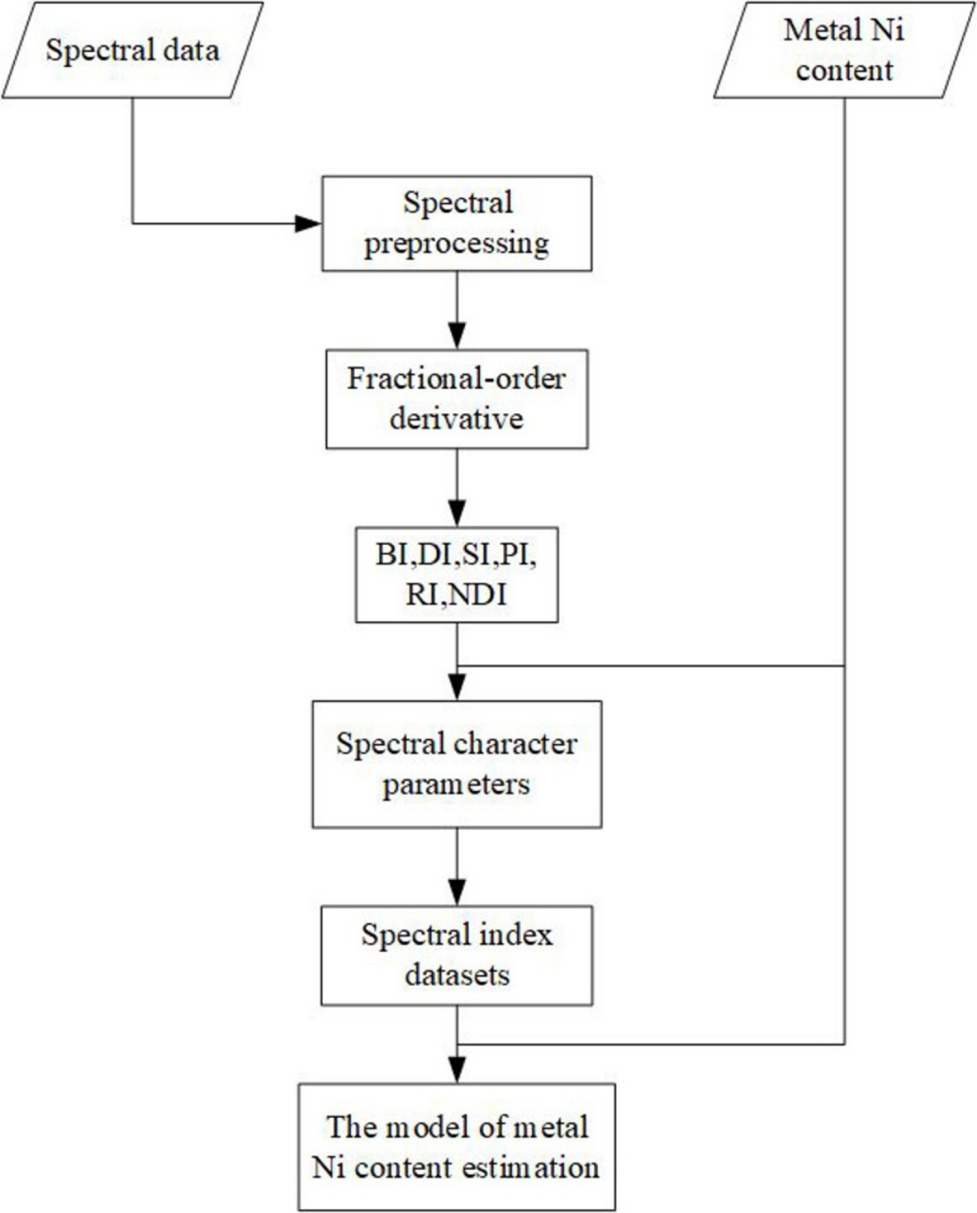
The overall workflow of the method for detection of Ni in soil.

The methodology workflow of utilizing prediction of nickel concentration in soil using fractional-order derivative and visible-near-infrared spectroscopy indices was shown in Fig 3. To improve the correlation between the spectral data and the Ni concentration, FOD was used for spectral signal processing. Then, six spectral indices were selected as the independent variable. Finally, PLSR models were established to estimate the Ni content of the soil.

#### 2.4.1 Fractional-order derivative

A spectral derivative algorithm is commonly used in preprocessing methods to eliminate the baseline drift. The FOD is a method of differentiation that extends the traditional integer differential to any order. The concept of the FOD was first recorded in the correspondence of two mathematicians Leibniz and L ’hospital [32] and has been applied in many fields. Compared with the traditional integer derivative, the FOD can completely mine the details of the spectral information. The spectral data conversion process is improved compared to the use of only first-order slope information and second-order conversion rate information. By obtaining the details of the changes in the spectral data, more correlation information about the weak Ni signal can be obtained in order to improve the correlation between the spectral data and the Ni concentration and the prediction accuracy of the estimation model of the Ni content. The commonly used FOD algorithms mainly include the Grunwald-Letnikov fractional calculus, Riemann-Liouville calculus, and Caputo fractional derivative algorithms [21]. The Grunwald-Letnikov fractional calculus algorithm was used in this study. This algorithm is suitable for spectral signal processing, and it can be considered to be an extension of the numerical sequence difference range of the form. The generalized expression of the Grunwald-Letnikov fractional calculus algorithm is

$$\begin{array}{l} \frac{d^{v}f(x)}{dx^{v}}\approx f(x)+(-v)f(x-1)+\\ \frac{\left(-v)(-v+1\right)}{2}f(x-2)+\cdots +\\ \frac{\tau (-v+1)}{n|\tau (-v+n+1)}f(x-n), \end{array}$$ (1)

where *x* is the reflectivity of the corresponding band, *v* is the order of the fractional-order derivative, *τ* (-*v*+*n*+1) is the Gamma function, and *n* is the difference between the upper and lower derivatives. Matlab software R2015b was used to calculate FOD.

#### 2.4.2 Spectral indices

The Vis-NIR spectrum is characterized by a wide spectrum coverage and a high spectral resolution. Spatial correlation, spectral correlation, and other interferences between adjacent bands lead to a great deal of data redundancy in the spectrum, which affects the extraction of the sensitivity data correlated to Ni in soil. To improve the correlation between the spectral information and the Ni signal and to reduce the redundancy of the spectral data, six spectral indices, i.e., the Brightness Index (BI), Normalized Difference Index (NDI), Ratio Index (RI), Difference Index (DI), Product Index (PI), and Sum Index (SI), were selected in order to extract the sensitivity data from the spectral data of different orders. On the basis of the sensitivity data extracted, the first four band combinations of the correlation coefficient based on each index were selected to form the index dataset to construct the estimation model of Ni content. The equations for calculating the six spectral indices are presented in Table 1. Matlab software R2015b was used to calculate spectral indices.

**Table 1:** The calculation formula of six spectral indices.

Spectral indices	Formula
Brightness index	$BI=\sqrt{\left(R1^{2}+R2^{2}\right)}$
Normalized difference index	*NDI(R*1, *R*2) = (*R*1 − *R*2) / (*R*1 + *R*2)
Ratio index	*RI*(*R*1, *R*2) = *R*1 / *R*2
Difference index	*DI*(*R*1, *R*2) = *R*1 − *R*2
Product index	*PI*(*R*1, *R*2) = *R*1 * *R*2
Sum index	*SI*(*R*1, *R*2) = *R*1 + *R*2

In the equations in Table 1, R1 and R2 are the reflectances of the two wavelengths in the range of 401–2400 nm.

#### 2.4.3 Statistical modeling

The partial least squares regression (PLSR) model was used to estimate the nickel content of the soil. PLSR is a corrective mathematical algorithm that can analyze spectral data in the presence of noise, collinearity, and other disturbances. It can extract the composite spectral variables with the strongest ability to explain the Ni content of the soil, reduce the data dimension, and improve the accuracy of the model [33]. In some studies, single-band spectral data have been used as a variable to study heavy metals in soil [34, 35], but the correlation between the spectrum and heavy metal contents was weaker in the areas with low degrees of pollution. Vis-NIR spectral estimation model of the Ni concentration of soil was established using best spectral index sets. During the regression modeling, the spectral index sets were set as the independent variables, and the Ni content was set as the dependent variable. The BI, NDI, RI, DI, PI, and SI were compared. IBM SPSS Statistics26 was used to perform PLSR.

#### 2.4.4 Verification of model accuracy

The 108 datasets were divided into a calibration set and verification set using the K-S method, the rationality of the samples and the reliability of the models was improved. The predictive ability of the model was assessed using three statistical indicators: the determination coefficient (*R*^2^), root mean square error (RMSE), and residual predictive deviation (RPD). *R*^2^ represents the percentage of all of the estimated values that can be interpreted using the spectral information. A larger *R*^2^ value indicates a stronger correlation between the estimated Ni concentration and the spectral data and a better prediction effect. The RMSE represents the degree of deviation of the estimated value from the real value. The smaller the RMSE is, the closer the estimated Ni content is to the measured content, and the more stable the model is. The RPD represents the prediction ability of the Ni hyperspectral estimation model, and the larger RPD is, the more reliable the model’s estimation is. A larger *R*^2^, smaller RMSE, and larger RPD indicates stronger modeling and a higher reliability. In terms of the RPD alone, the prediction ability of the model is not ideal when RPD < 1.4, the prediction ability of the model is average when 1.4 < RPD < 2.0, and the model’s prediction ability improves when RPD > 2.0 [36].

## 3. Results

### 3.1 Characteristic amount of Ni in soil

The statistical results of the Ni contents of the 108 soil samples used in this study are presented in Table 2. The Ni content ranged from 12.32 mg·kg^-1^ to 74.61 mg·kg^-1^, with an average of 28.83 mg·kg^-1^. The average Ni content was higher than the background value (25.3 mg·kg^-1^) of the local soil, and the maximum value was 3.17 times that of the background value. The statistical characteristics of the entire, calibration, and validation datasets were similar, indicating the rational selection of the calibration dataset and validation dataset and their strong data representation ability. The coefficients of variation of the entire, calibration, and validation datasets all ranged from 16% to 35%, i.e., moderate variation [37]. The good sample grouping had a positive effect on the later model optimization.

**Table 2:** Characteristic amount of Ni in soil for the entire, calibration, verification datasets.

Statistics	Entire dataset	Calibration dataset	Verification dataset
Observation	108	76	32
Maximum (mg·kg^-1^)	74.61	74.61	33.31
Minimum (mg·kg^-1^)	12.32	12.32	17.91
Mean (mg·kg^-1^)	28.83	30.29	25.69
SD (mg·kg^-1^)	8.33	9.36	4.32
CV (%)	28.89	30.91	16.84

### 3.2 Fractional-order derivative spectra curves

Similar to previous studies, the reflectance of the original spectrum in the visible band (400–800 nm) gradually increased at a rapid rate, and it began to slow down and gradually stabilized in the near-infrared band. There were three obvious absorption valleys in the original spectrum at 1400 nm, 1900 nm, and 2200 nm, which were mainly related to the absorption characteristics of water molecules [38]. The concentration of Ni in the soil could not be interpreted and estimated well due to the baseline drift and overlapping peaks in the original spectral curve.

**Fig 4:**
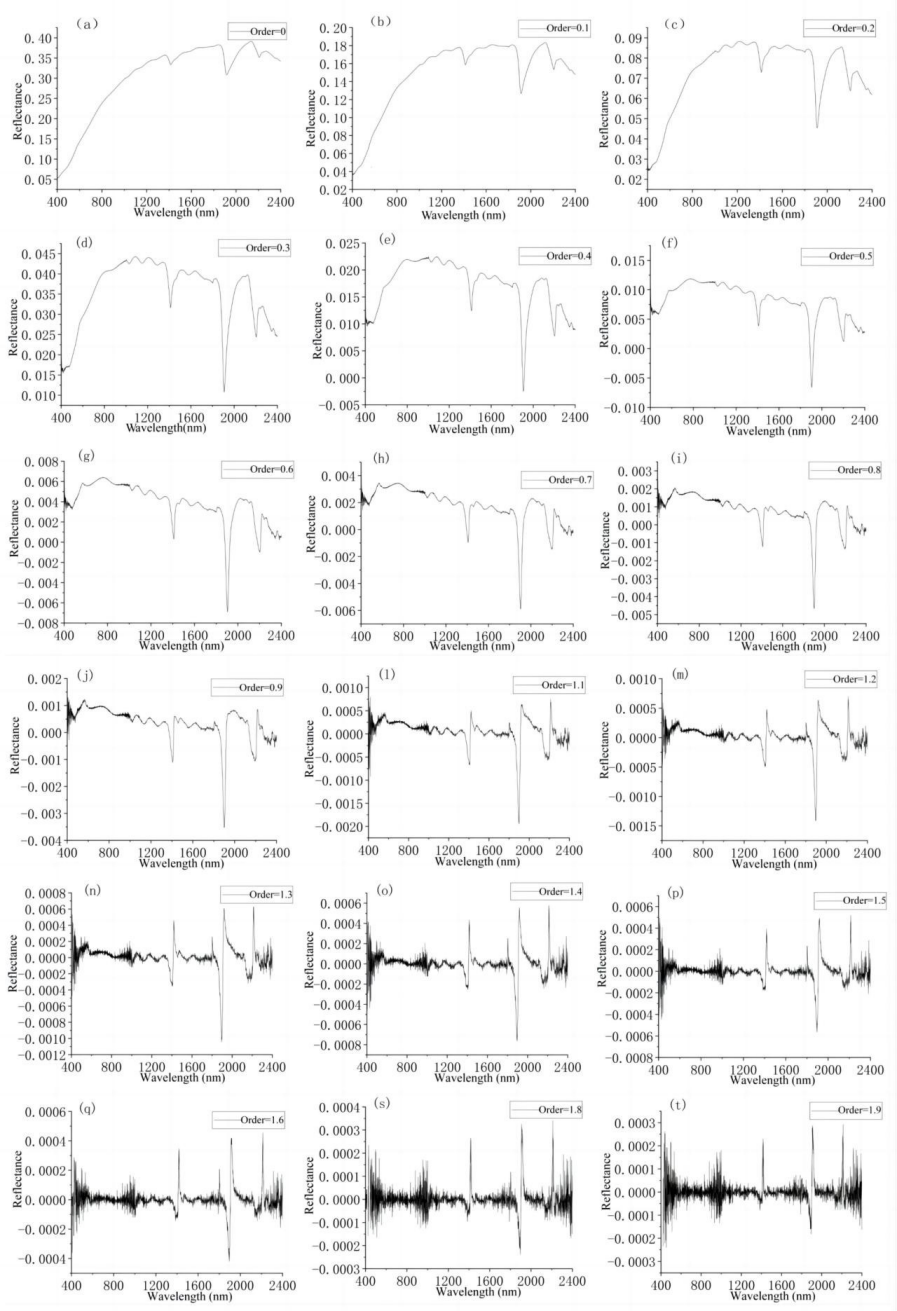
FOD spectra of the calibration dataset: original(a), 0.1-order(b), 0.2-order(c), 0.3-order(d), 0.4-order(e), 0.5-order(f), 0.6-order(g), 0.7-order(h), 0.8-order(i), 0.9-order(j), 1.0-order(k), 1.1-order(l), 1.2-order(m), 1.3-order(n), 1.4-order(o), 1.5-order(p), 1.6-order(q), 1.7-order(r), 1.8-order(s), 1.9-order(t), and 2.0-order(u).

The mean FOD spectral curves are shown in Fig 4B–4U. As the FOD increased, the interference of the baseline drift was eliminated with decreasing reflection intensity, and the overlapping peaks were removed as more absorption valleys and reflectance peaks appeared. During the change from order 0 to order 1, the three absorption valleys related to water molecule absorption became sharper, and reflection peaks appeared at 1400 nm, 1900 nm, and 2200 nm. In addition, there was a small negative valley near 490 nm and a small positive peak near 550 nm, which were mainly due to the hematite and iron oxides in the soil [39, 40]. More absorption valleys and reflection peaks appeared when the order increased from 1 to 2. At 1400 nm, the absorption valley decreased gradually and the reflection peak became sharper. The absorption valley and the reflection peak at about 1900 nm became very sharp. After order 1.5, it was difficult to effectively distinguish the characteristic changes in the spectral curves. Spectral curve of order 1 to order 2 had more features and noise than that of order 0 to order 1.

These results demonstrate that preprocessing of the spectral data using the FOD can not only eliminate the influence of the baseline drift and overlapping peaks but also reveal more characteristics. It is helpful to extract the sensitivity data correlated to the Ni content of the soil.

### 3.3 Correlation of Ni to spectral indices

**Fig 5:**
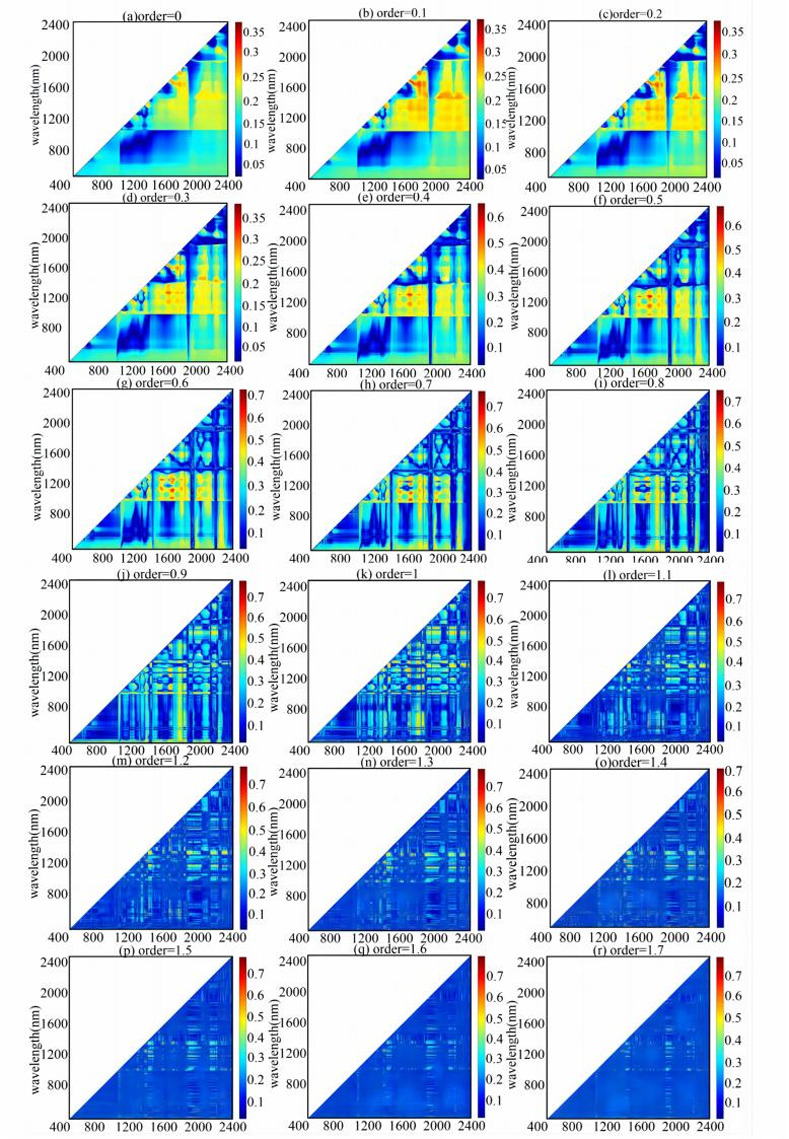
Correlation between the NDI and Ni content for different orders: 0-order(a), 0.1-order(b), 0.2-order(c), 0.3-order(d), 0.4-order(e), 0.5-order(f), 0.6-order(g), 0.7-order(h), 0.8-order(i), 0.9-order(j), 1.0-order(k), 1.1-order(l), 1.2-order(m), 1.3-order(n), 1.4-order(o), 1.5-order(p), 1.6-order(q), 1.7-order(r), 1.8-order(s), 1.9-order(t), and 2.0-order(u).

The correlations between the BI, RI, DI, PI, SI, and NDI and the Ni content of the soil were analyzed for different fractional-orders. The sensitivity data was extracted to construct the spectral index datasets. The correlation between the NDI and the Ni content is shown in Fig 5, and the correlations between the BI, RI, DI, PI, and SI and the Ni content are shown in Figure S. 1-S. 5 (presented in S1 Appendix). The color band on the right represents the correlation coefficient (r), and the X-axis and Y-axis represent the spectra with wavelengths of 401 nm and 2400 nm, respectively.

The correlation between the spectral index and the Ni content changed with the FOD. The maximum correlation coefficient was only 0.358 for order 0 (Fig 3), and it gradually increased with increasing fractional order. The correlation coefficient reached the maximum at order 1.9 (r = 0.804, Fig 3) and then decreased slightly. The band combinations with high correlations were mostly located in the near-infrared region at low fractional orders (orders 0 to 1). It was difficult to intuitively identify the combination of sensitivity bands due to the gradual thinning of the spectral features and the increasing noise at high fractional orders (orders 1.1 to 2). This is consistent with the conclusion shown in Fig 4, i.e., that the spectral characteristics were enhanced and noise appeared with increasing fractional order. The fractional order of the combination of spectral bands with the maximum correlation varied under different spectral indices. The fractional order of the NDI band combination with the greatest correlation was order 1.9 (r = 0.804), followed by order 1.6 (r = 0.782). In contrast, the maximum correlations of the original reflectance (r = 0.358), first-order reflectance (r = 0.759), and second-order reflectance (r = 0.764) were lower. This further indicates that compared with the original spectra and conventional integer derivatives, the FOD improved the correlation between the spectral data and the micro-signals of the Ni content of the soil. The first four NDIs of the correlation coefficient were selected from Fig 5, and the first four BIs, PIs, DIs, RIs, SIs were selected from Fig 5S. The spectral index datasets of the NDIs, BIs, PIs, DIs, RIs, SIs were presented in Table 3.

**Table 3:** The best four indices of BI, PI, DI, RI, SI and NDI extracted from different fractional-order derivatives (FOD) spectra.

	(Combination) order	(Combination) order	(Combination) order	(Combination) order
BI	(R_1659_, R_1732_) 1.4	(R_1732_, R_2088_) 1.3	(R_536_, R_2226_) 2.0	(R_491_, R_825_) 2.0
PI	(R_900_, R_1298_) 1.6	(R_2179_, R_2344_) 2.0	(R_639_, R_2001_) 1.9	(R_639_, R_2001_) 2.0
DI	(R_491_, R_2227_) 2.0	(R_2135_, R_2227_) 2.0	(R_2135_, R_2227_) 1.9	(R_1061_, R_2227_) 2.0
RI	(R_1490_, R_1614_) 2.0	(R_921_, R_1614_) 2.0	(R_1325_, R_1614_) 2.0	(R_962_, R_1497_)1.5
SI	(R_491_, R_2226_) 2.0	(R_2342_, R_2227_) 2.0	(R_2134_, R_2227_) 2.0	(R_2348_, R_2227_) 2.0
NDI	(R_709_, R_1631_) 1.9	(R_542_, R_1000_) 1.9	(R_404_,R_717_) 1.6	(R_1381_, R_1806_) 1.3

As can be seen from Table 3, the wavelengths of 1614, 1732, 2001, 2134, and 2227 nm were selected many times, suggesting that they may be particularly important in the estimation of the Ni content of soil.

### 3.4 Modeling and accuracy evaluation

The extracted index datasets and Ni contents were used to correct the PLSR model and to evaluate its accuracy. The accuracy evaluation results are presented in Table 4. Among the six models, the models based on the RI dataset ($R_{CV}^{2}$ = 0.700, $R_{P}^{2}$ = 0.679, RMSEC = 4.561, RMSE = 4.588) and the NDI dataset ($R_{CV}^{2}$ = 0.745, $R_{P}^{2}$ = 0.735, RMSEC = 3.629, RMSE = 3.848) had the best performances and a high stability. The RPD values of the models based on the BI dataset (1.299) and DI dataset (1.260) were less than 1.4, indicating poor model reliability. The RPD values of the models based on the PI dataset (1.560) and SI dataset (1.540) were between 1.4 and 2, indicating average model reliability. The RPD values of the RI dataset (2.032) model and the NDI dataset (2.423) model were greater than 2, indicating that these models had a high reliability. In contrast, the maximum RPD of the model based on the index dataset constructed from the original spectral data (only 1.176) was lower than the modeling accuracy of the other spectral index datasets.

**Table 4:** Accuracy evaluation for the estimation of Ni in soil using the method of fractional-order derivative (FOD) and spectral indices.

Index	Calibration dataset		Validation dataset		
	$R_{CV}^{2}$	RMSEC (mg·kg^-1^)	$R_{P}^{2}$	RMSE (mg·kg^-1^)	RPD
BI	0.492	5.935	0.277	7.177	1.299
PI	0.600	5.268	0.508	5.976	1.560
DI	0.405	6.424	0.235	7.401	1.260
RI	0.700	4.561	0.679	4.588	2.032
SI	0.464	6.101	0.485	6.055	1.540
NDI	0.745	3.629	0.735	3.848	2.423

**Fig 6:**
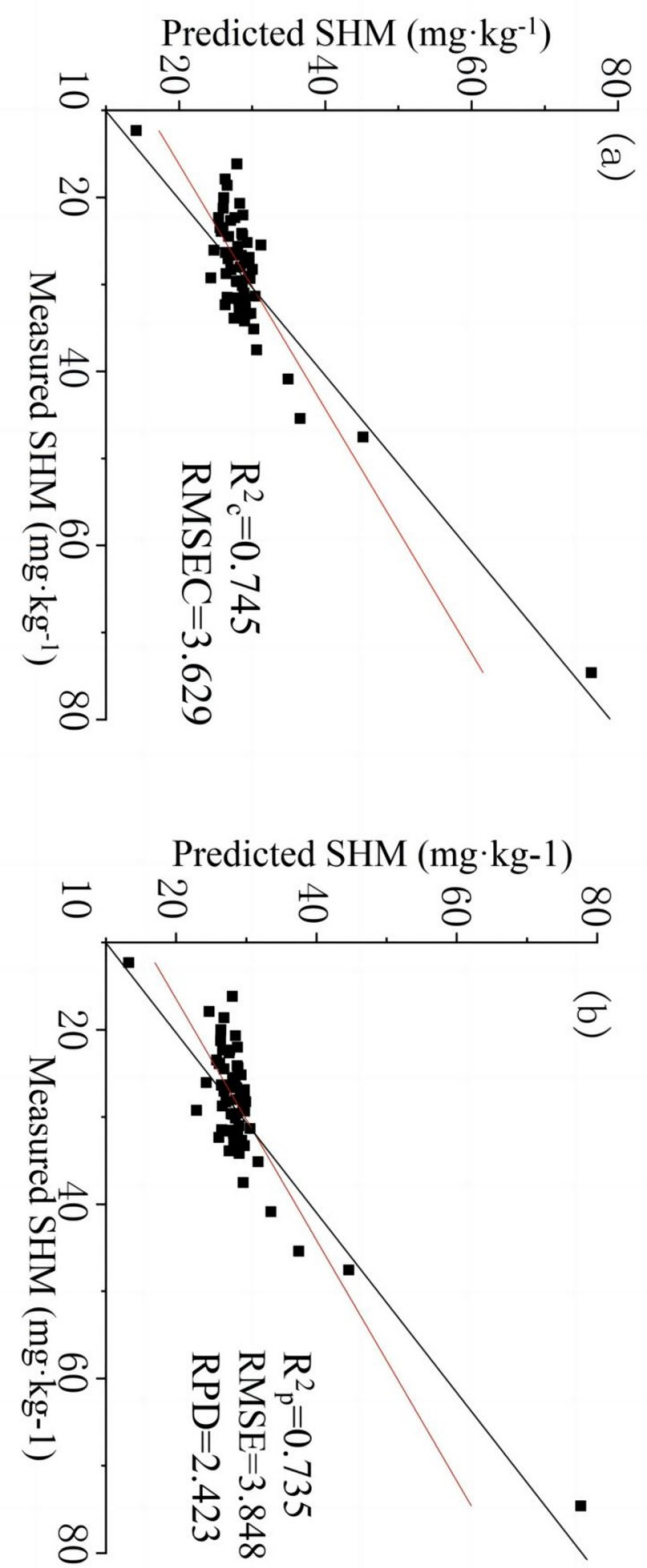
Scatter plots of the predicted Ni content in soil based on the calibration set(a) and validation set(b) with the NDI dataset. Black solid lines indicate the 1:1 lines.

The results show that using the spectral index datasets extracted from the FOD spectra effectively improved the accuracy and stability of the model. Prediction scatter diagrams of the modeling dataset and verification dataset based on the NDI dataset are shown in Fig 6.

## 4. Discussion

Vis-NIR spectroscopy is an economical, rapid, and convenient method of estimating and inverting the Ni content of soil. However, the difficulty of the estimation lies in the weak signal of Ni in soil and its low correction with the spectral data. In this study, the method of combining the FOD and the spectral index was developed as a new way to extract the Vis-NIR spectral information and estimate the Ni content of soil. The FOD preprocessing of Vis-NIR spectral data can eliminate the baseline drift and overlapping peaks of the original spectral curve and can detect the characteristic information more efficiently [41–45].

Previous studies on heavy metals in soil have used processing methods such as logarithmic transformation and first- and second-order derivation transformation based on spectral data in combination with PCA, SVM, GWR, and other methods to establish heavy metal estimation models [14, 15]. However, these methods of data processing do not take the details of the processing of the change in the spectral information into account, so some of the useful information is lost. FOD is a new technique that was recently introduced into the field of remote sensing, and it plays a significant role in the extraction of spectral information and can detect the details of spectral information changes [38, 43]. As can be seen from Fig 3, the spectral data exhibit more characteristics for the non-integer orders. As the fractional order increases, new wave peaks and absorption valleys continuously appear, but the noise also increases gradually. In order to reduce the influence of the noise and efficiently extract the useful information, we extracted the sensitivity data correlated to the Ni content of the soil using a combination of the FOD and spectral index. As can be seen from Fig 5, the maximum correlations between the NDI and the Ni content occurred for non-integer orders, i.e., order 1.9 (r = 0.804, r = 0.786), order 1.6 (r = 0.782), and order 1.3 (r = 0.775). The spectral index algorithm can consider the interaction between bands effectively, reduce the influence of noise, and avoid the phenomenon of over-fitting.

The PLSR can extract important information from a large number of data to reduce the data dimension, reduce the computing costs, and improve the accuracy of the estimation. Six estimation models were constructed based on the spectral index datasets extracted from the FOD spectra, and one was constructed based on the spectral index dataset extracted from the original spectra. It was found that there were differences among the models. The models based on the NDI and RI had the best effect, with RPD values of 2.423 and 2.032, respectively, while the RPD of the model based on the original spectral data was only 1.176, indicating a great difference in the model reliability.

The RMSEC and RMSE of the model based on the NDI dataset extracted from the FOD spectra were decreased from 7.772 and 8.143 to 3.629 and 3.848, respectively, and the $R_{CV}^{2}$ and $R_{P}^{2}$ were increased from 0.134 and 0.088 to 0.745 and 0.735, respectively. The experimental results show that the method that combines the FOD and spectral index greatly improved the accuracy and stability of the estimation model of the Ni content, and thus, it provides a new direction for estimating the heavy metal contents of soil in the future.

The data in this study were measured for dry soil sifted through a 100 mesh sieve in order to eliminate the influences of the diameter of the soil particles, moisture, and other factors, so they differ from the spectral data collected in the field. In future studies, it will be necessary to overcome the influence of environmental factors on the soil spectrum.

## 5. Conclusions

In this study, the change in the spectral curve with the FOD was studied, and spectral indices were used to analyze the correlation between the spectral data and the Ni content of soil, finally, PLSR models were established to estimate the Ni content of the soil.

1. In the FOD pretreatment process, the spectra changed with increasing fractional order. The spectral detail were enhanced and more absorption valleys and reflectance peaks appeared, and the spectral reflectance decreased and gradually approached 0. The interference of the baseline drift and overlapping peaks was eliminated.
2. The method of combining the FOD with the spectral index effectively improved the correlation between the Vis-NIR spectra and the Ni content of the soil. After FOD treatment, the correlations of the six spectral indices with the Ni content increased and then decreased, but there were differences in the performances of the six spectral indices. The NDI had the best performance for order 1.9, with a correlation coefficient of 0.803.
3. The feasibility of the method of combining the FOD and the spectral index for the estimation of the Ni concentration was demonstrated. The performances of the estimation models based on six spectral index datasets constructed after FOD processing were better than that based on the original dataset. The estimation model based on the NDI dataset ($R_{P}^{2}$ = 0.735, RMSE = 3.848, RPD = 2.423) performed the best, and it had a great ability to estimate the Ni content of the soil in the study area.

The method of combining the FOD and the spectral index can extract the sensitivity data effectively and can improve the accuracy of the estimation of the Ni concentration.

## Supporting information

S1 Appendix Correlations between the BI, RI, DI, PI, and SI and the Ni for different orders. (DOCX)
