# Supplementary material for: Predicting nickel concentration in soil using fractional-order derivative and visible-near-infrared spectroscopy indices
Source: PLoS One. 2024 Aug 1;19(8):e0302420. doi: 10.1371/journal.pone.0302420 (PMC11293674; doi:10.1371/journal.pone.0302420)
Supplement: S1 Appendix — (DOCX) [file pone.0302420.s001.docx]

**Predicting nickel concentration in soil using fractional-order derivative and visible-near-infrared spectroscopy indices**

Jianfei Cao^a^, Wei Liu^b^*, Yongyu Feng^c^, Jianhua Liu^d^, Yuanlong Ni^b^

^a^ College of Geography and Environment, Shandong Normal University, Jinan 250014, China;

^b^ Shandong Yuanhong Survey Planing and Design CO.,LTD, Jinan 250014, China;

^c^ Shandong Provincial Institute Land Spatial Data and Remote Sensing Technology, Jinan 250001, China.

^d^ Jinan Institute of Surveying and Mapping, Jinan 250101, China.

* Correspondence: Liuwei: 15953166805@163.com

**
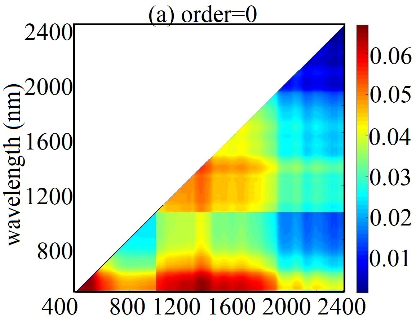

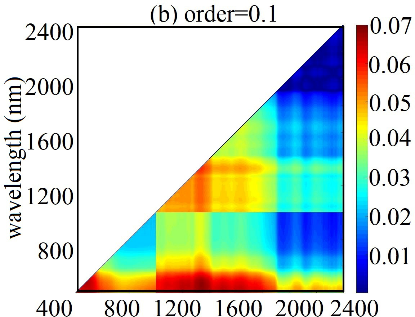

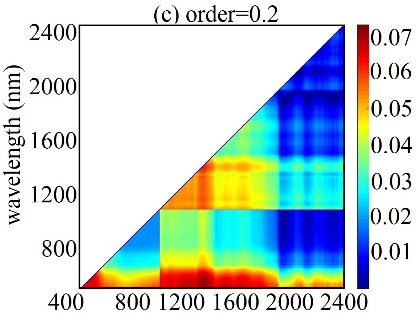
**

**
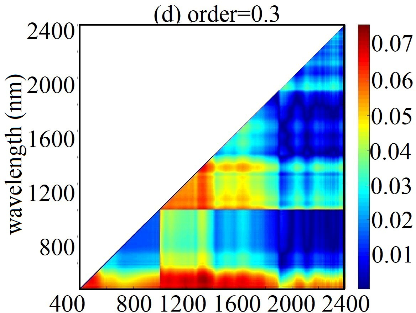

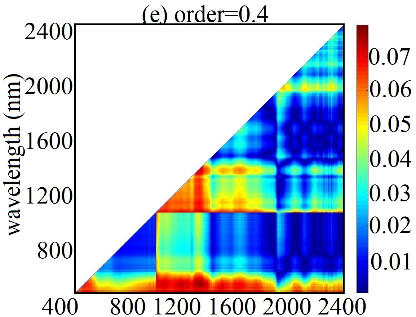

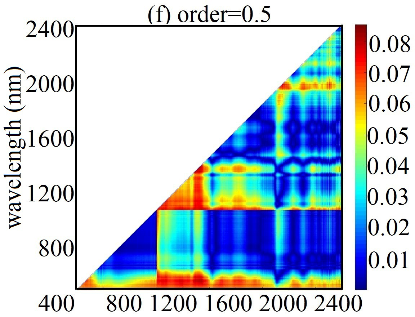
**

**
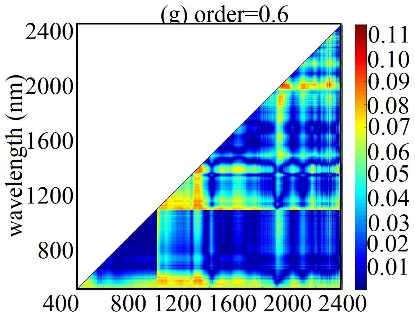

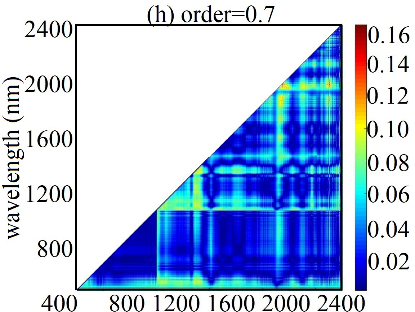

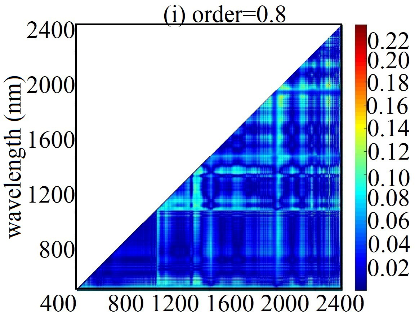
**

**
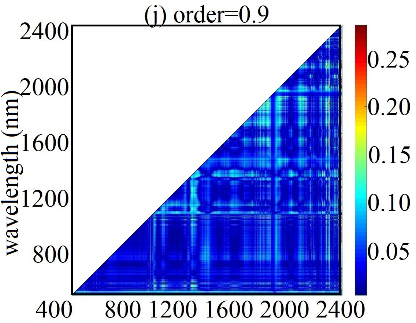

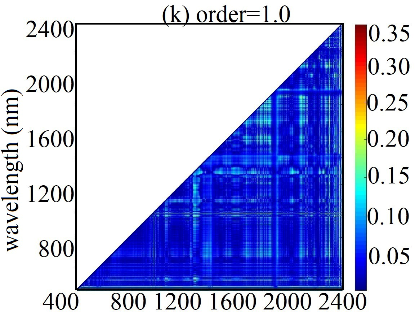

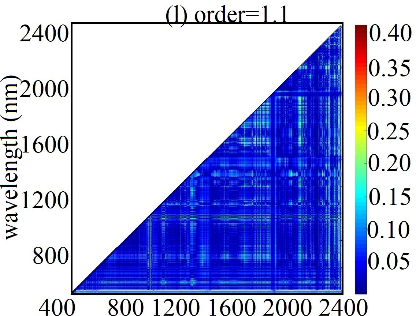
**

**
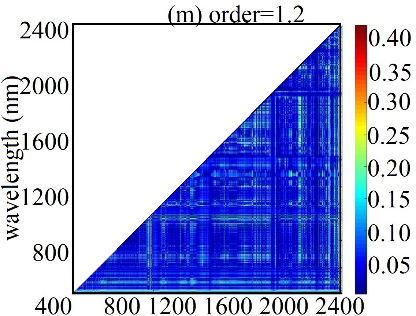

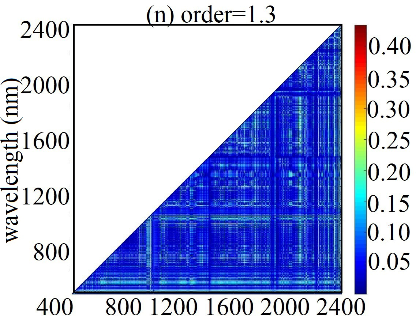

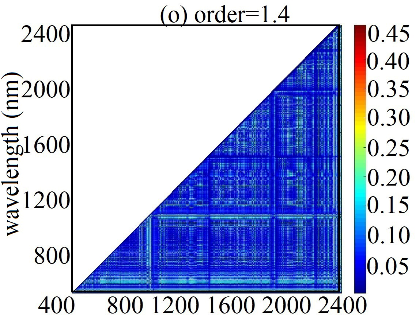
**

**
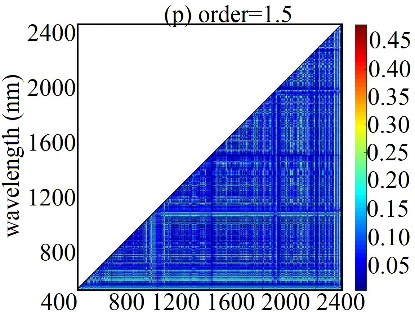

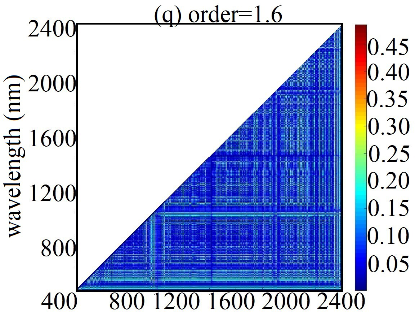

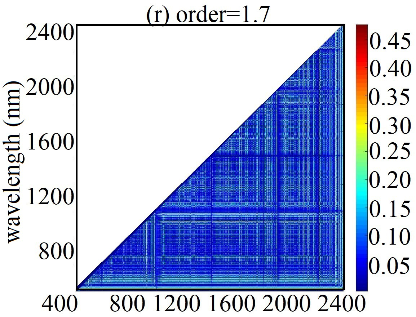
**

**
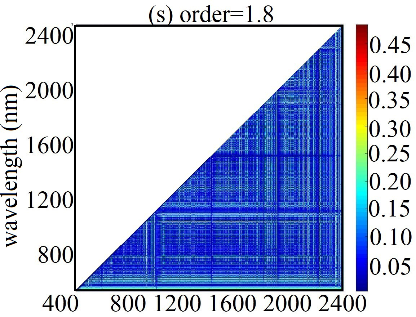

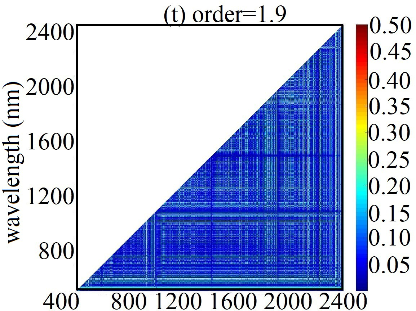

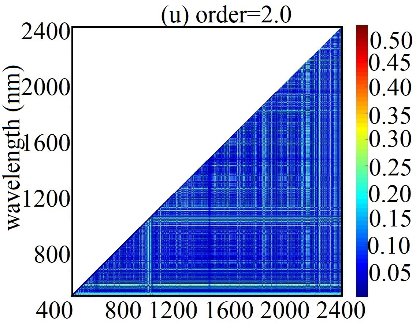
**

**Fig S1 Correlation between the Sum index (SI) and Ni content for different orders: 0-order(a), 0.1-order(b), 0.2-order(c), 0.3-order(d), 0.4-order(e), 0.5-order(f), 0.6-order(g), 0.7-order(h), 0.8-order(i), 0.9-order(j), 1.0-order(k), 1.1-order(l), 1.2-order(m), 1.3-order(n), 1.4-order(o), 1.5-order(p), 1.6-order(q), 1.7-order(r), 1.8-order(s), 1.9-order(t), and 2.0-order(u).**

**
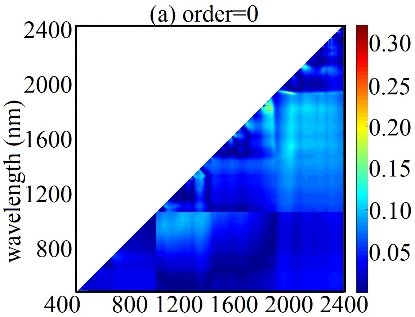

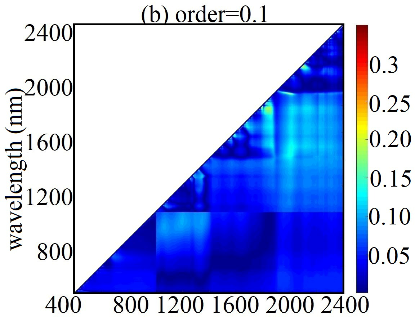

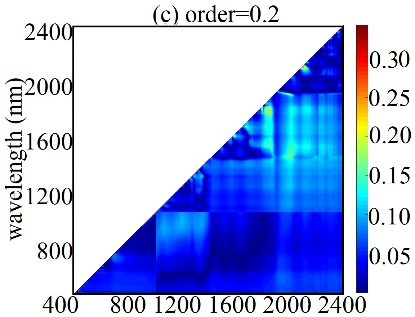
**

**
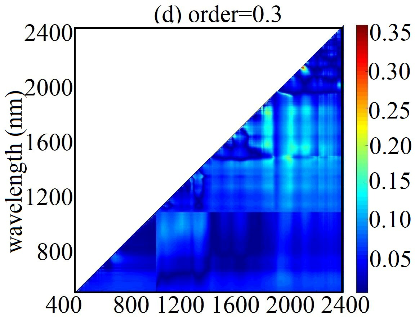

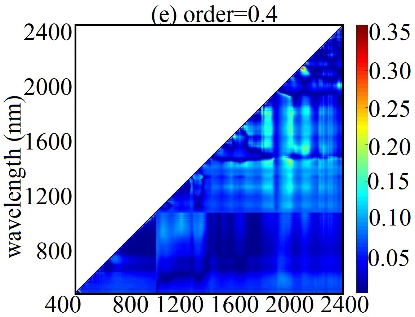

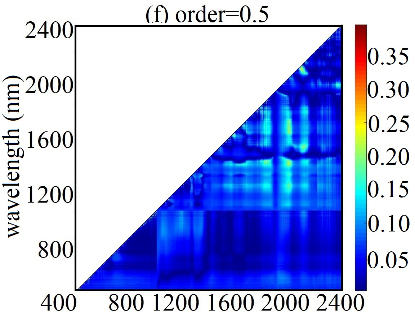
**

**
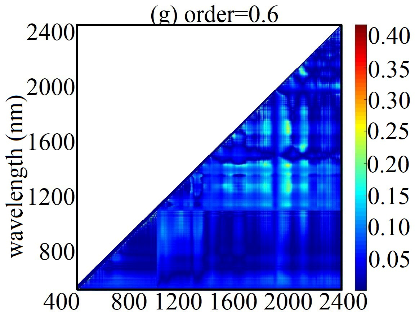

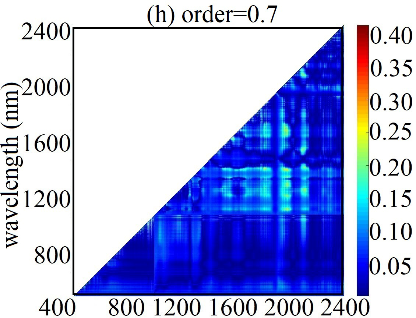

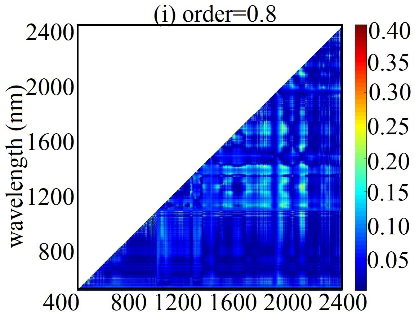
**

**
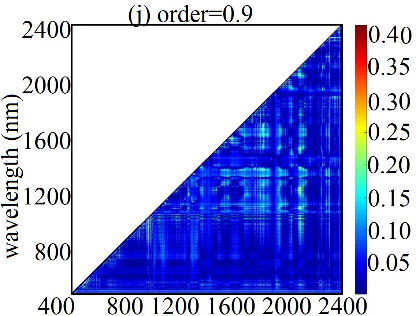

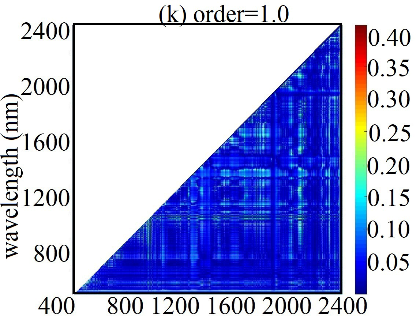

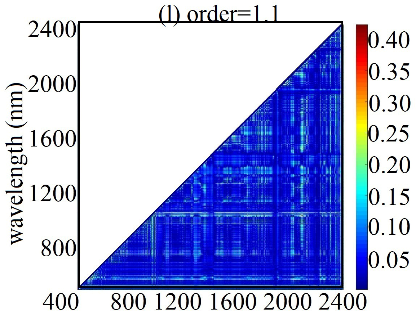
**

**
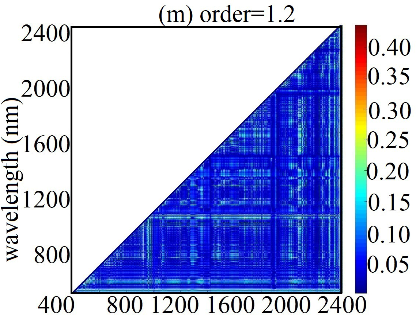

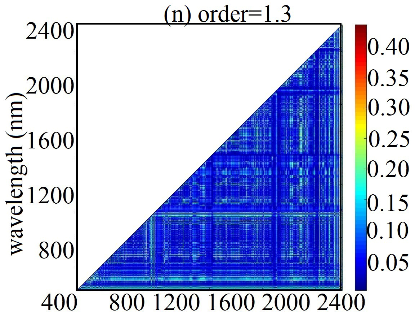

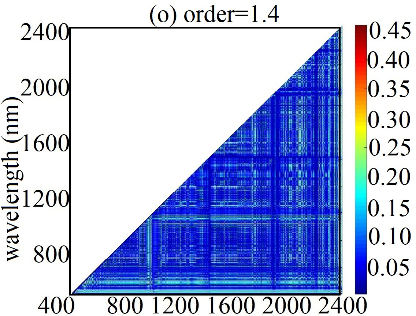
**

**
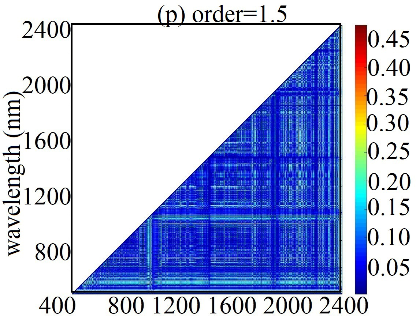

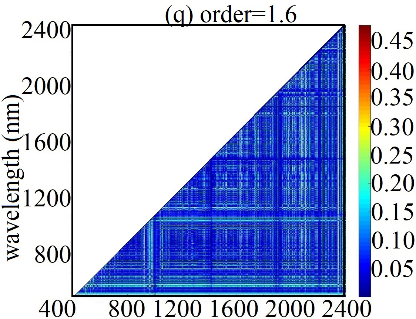

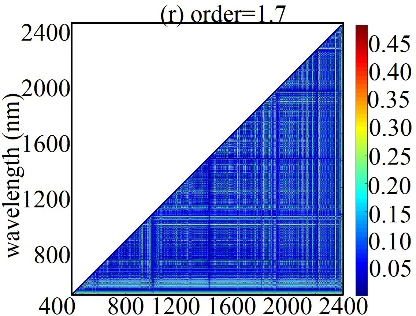
**

**
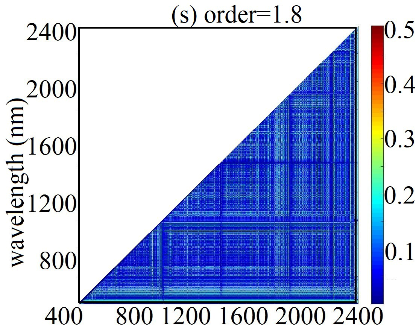

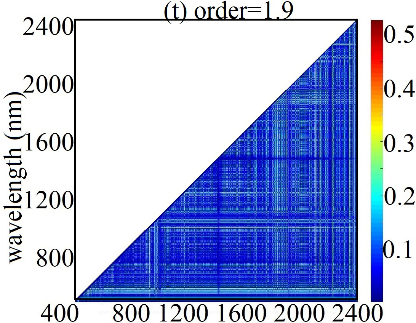

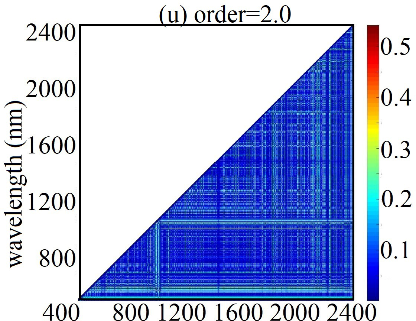
**

**Fig S2 Correlation between the Difference index (DI) and Ni content for different orders: 0-order(a), 0.1-order(b), 0.2-order(c), 0.3-order(d), 0.4-order(e), 0.5-order(f), 0.6-order(g), 0.7-order(h), 0.8-order(i), 0.9-order(j), 1.0-order(k), 1.1-order(l), 1.2-order(m), 1.3-order(n), 1.4-order(o), 1.5-order(p), 1.6-order(q), 1.7-order(r), 1.8-order(s), 1.9-order(t), and 2.0-order(u).**

**
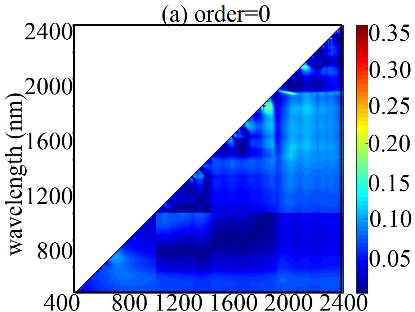

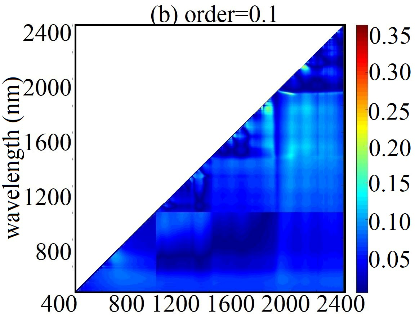

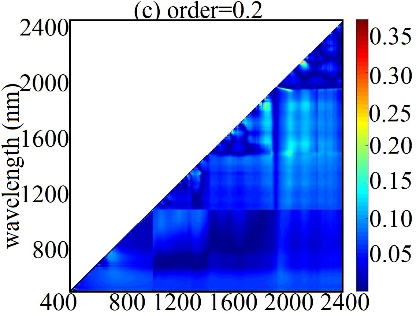

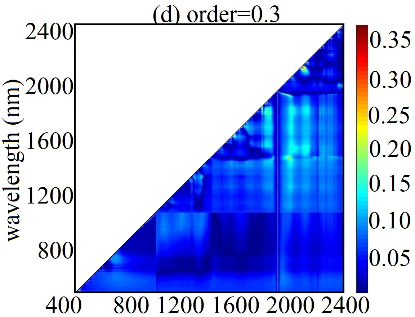

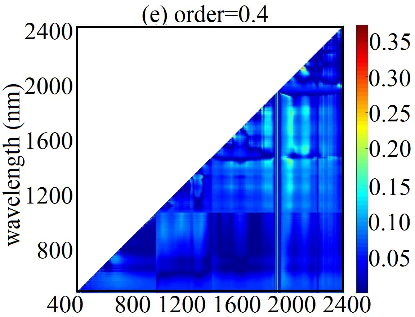

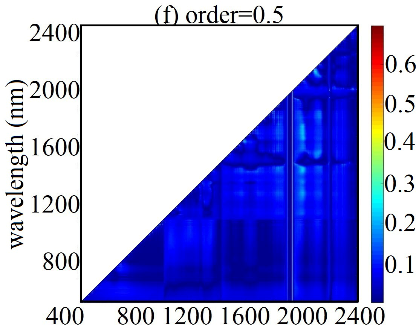

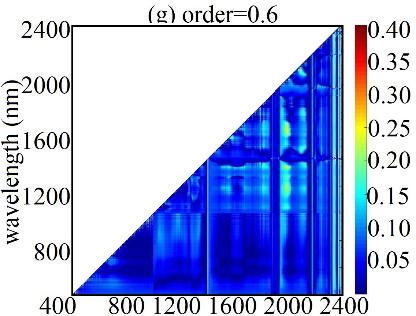

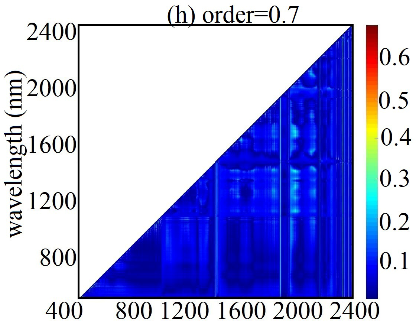

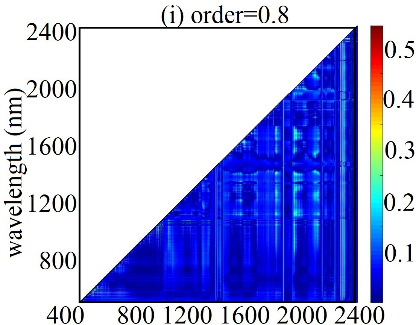

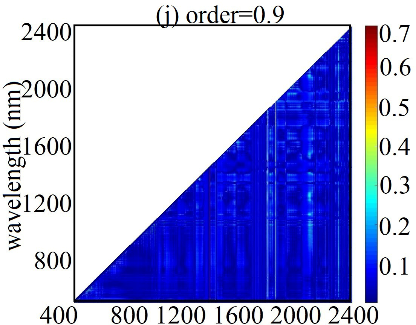

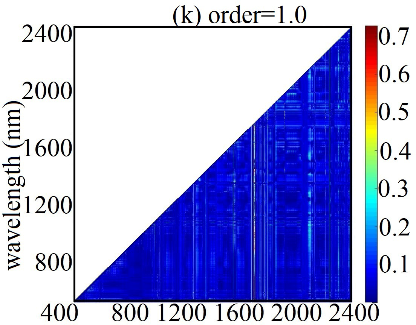

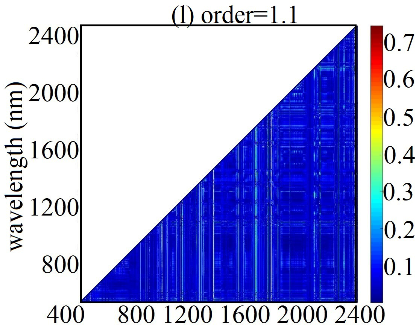

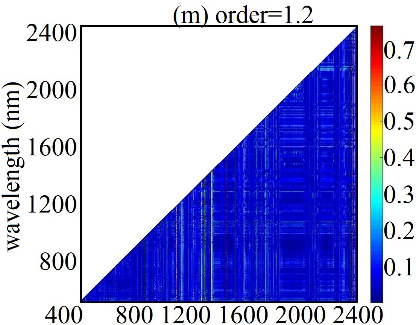

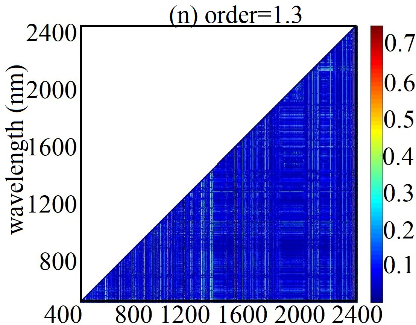

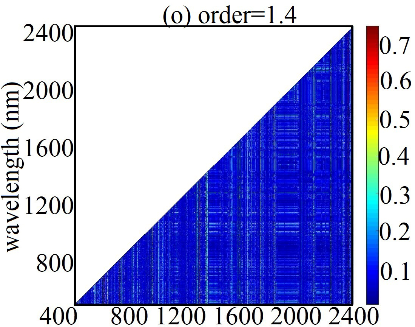

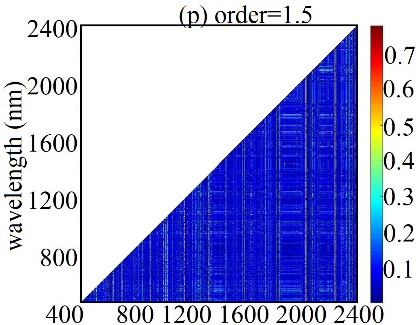

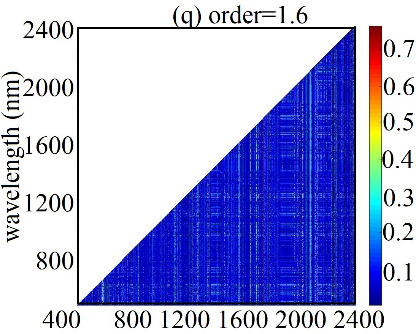

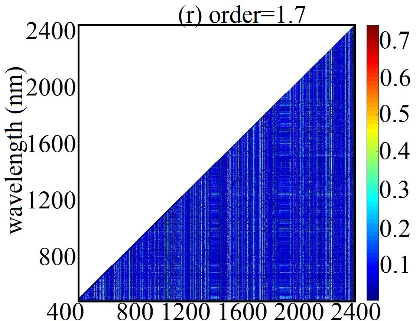

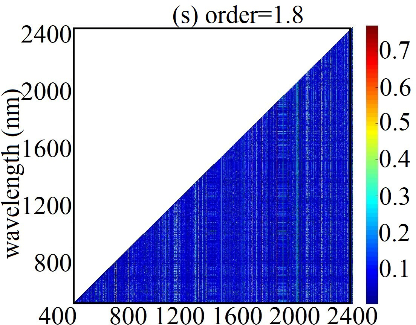

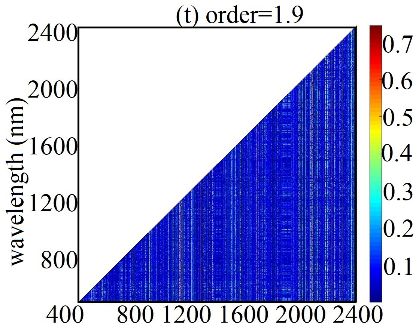

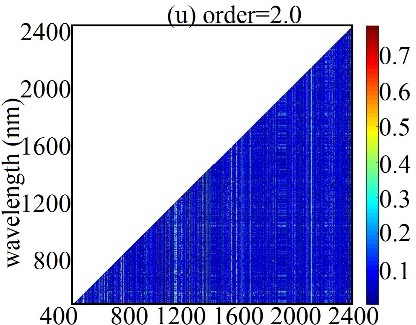
**

**Fig S3 Correlation between the Ratio index (RI) and Ni content for different orders: 0-order(a), 0.1-order(b), 0.2-order(c), 0.3-order(d), 0.4-order(e), 0.5-order(f), 0.6-order(g), 0.7-order(h), 0.8-order(i), 0.9-order(j), 1.0-order(k), 1.1-order(l), 1.2-order(m), 1.3-order(n), 1.4-order(o), 1.5-order(p), 1.6-order(q), 1.7-order(r), 1.8-order(s), 1.9-order(t), and 2.0-order(u).**


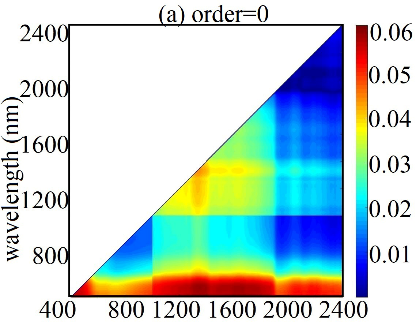

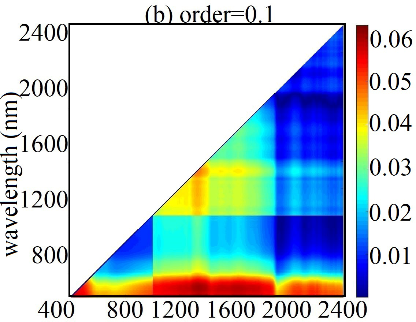

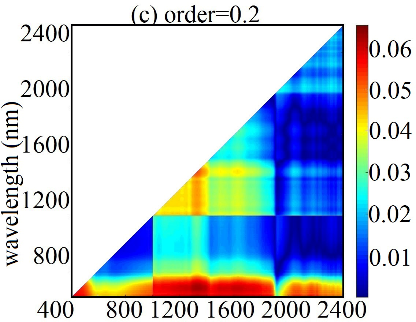

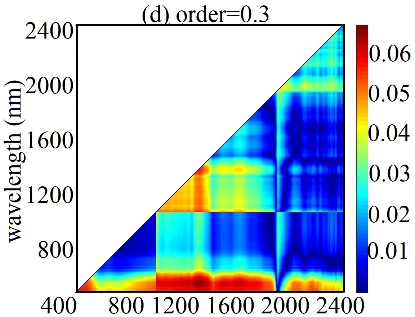

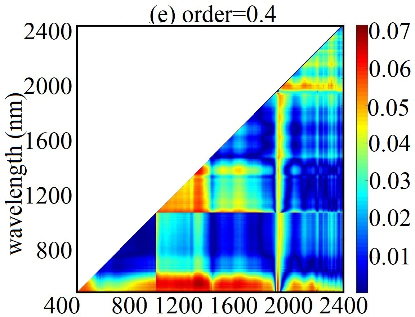

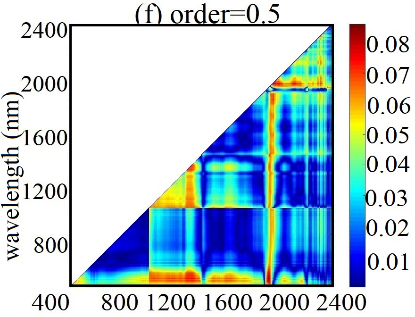

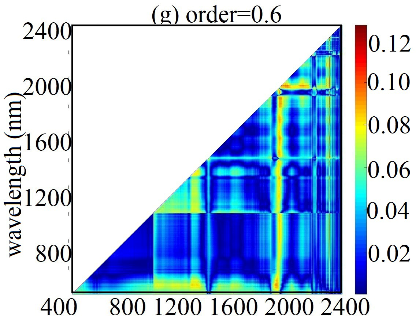

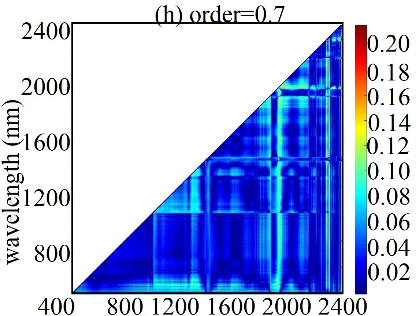

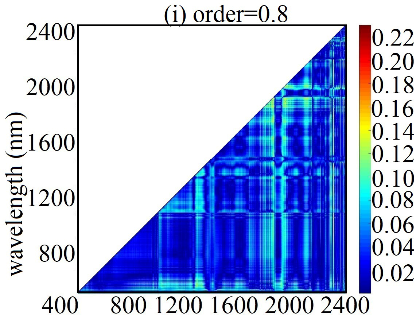

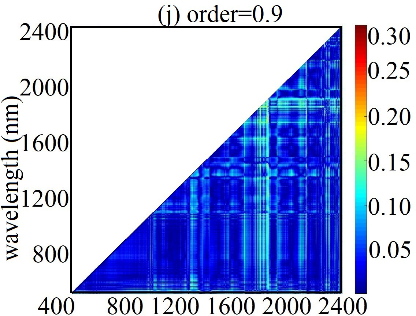

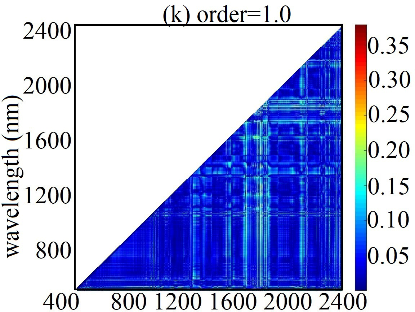

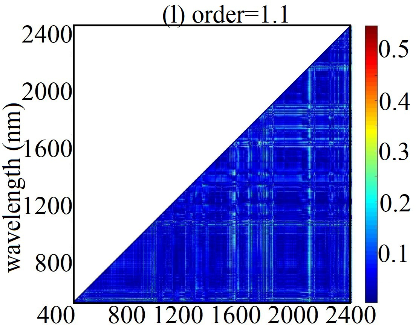

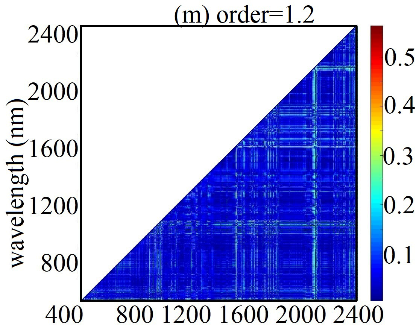

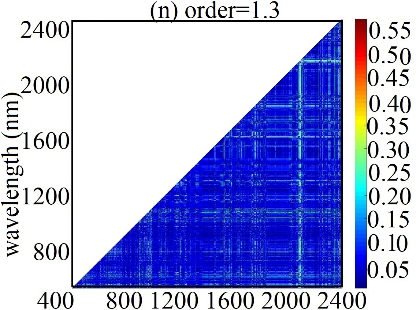

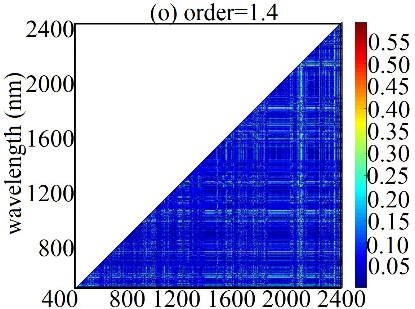

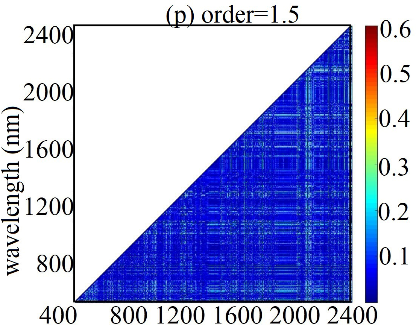

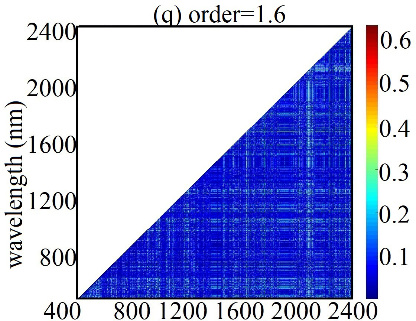

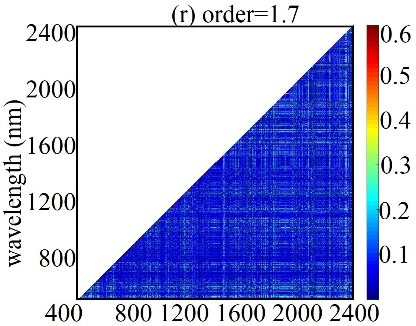

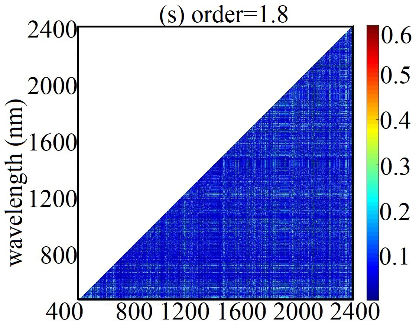

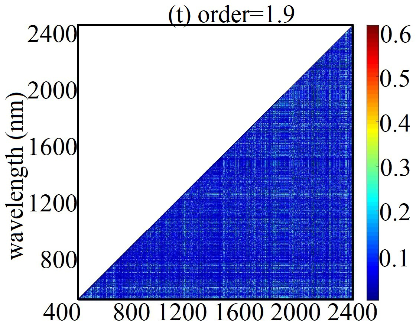

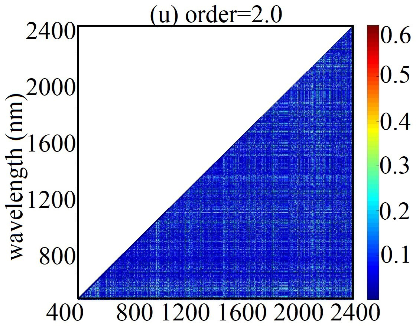


**Fig S4 Correlation between the Product index(PI) and Ni content for different orders: 0-order(a), 0.1-order(b), 0.2-order(c), 0.3-order(d), 0.4-order(e), 0.5-order(f), 0.6-order(g), 0.7-order(h), 0.8-order(i), 0.9-order(j), 1.0-order(k), 1.1-order(l), 1.2-order(m), 1.3-order(n), 1.4-order(o), 1.5-order(p), 1.6-order(q), 1.7-order(r), 1.8-order(s), 1.9-order(t), and 2.0-order(u).**


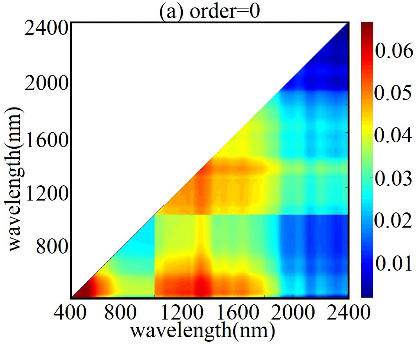

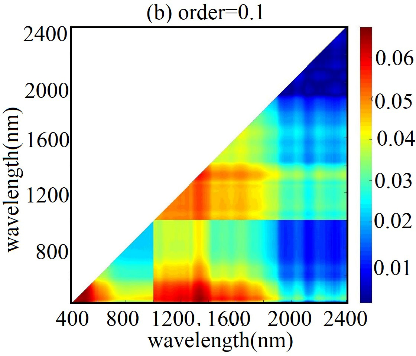

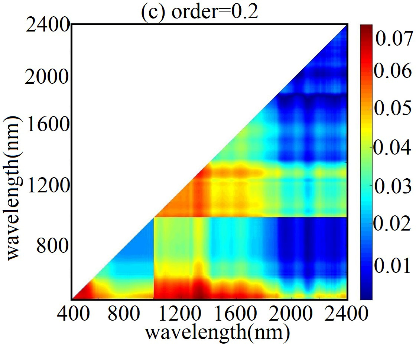

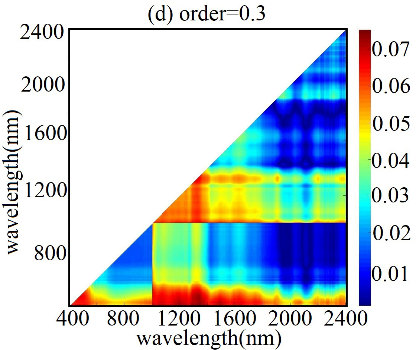

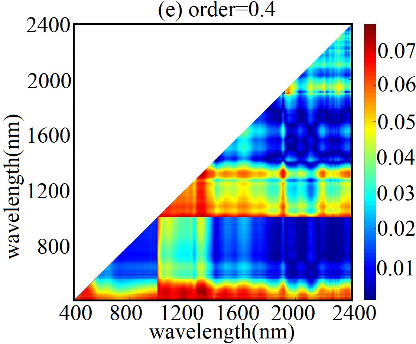

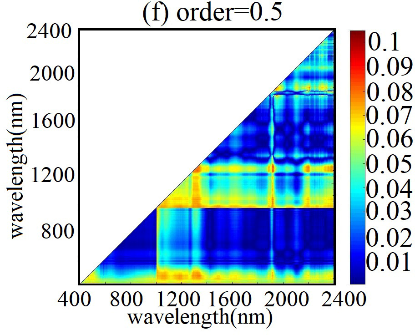

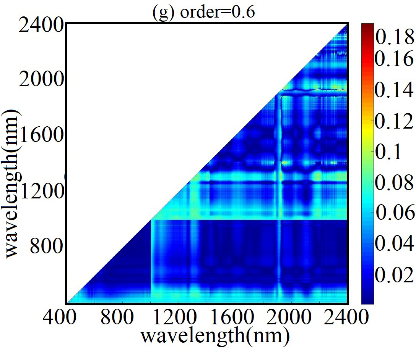

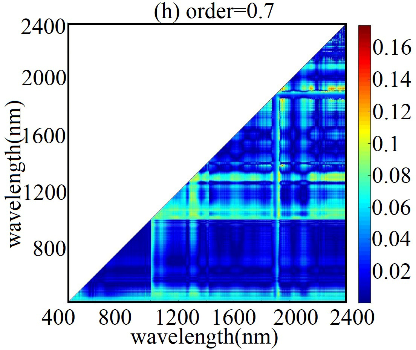

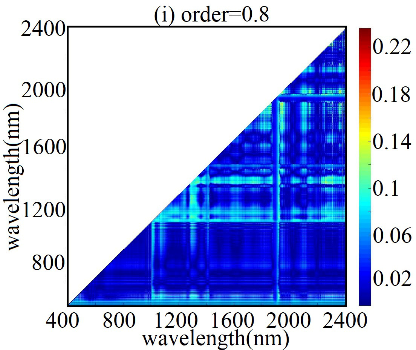

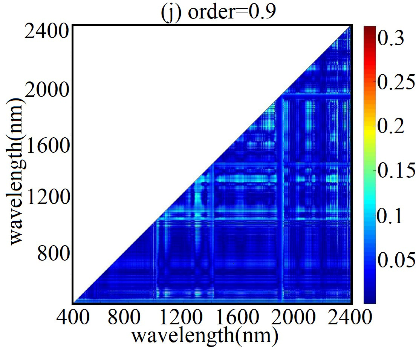

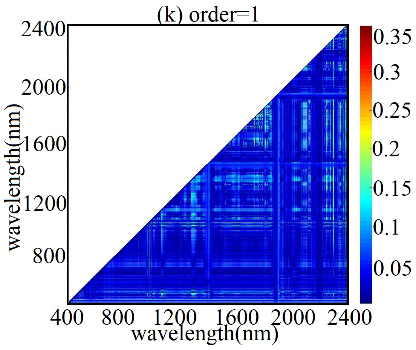

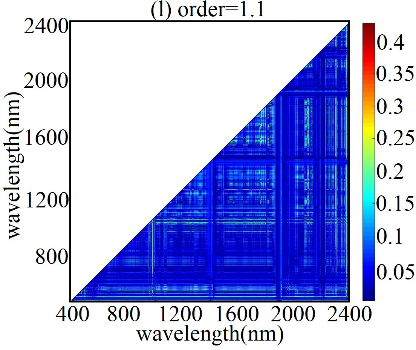

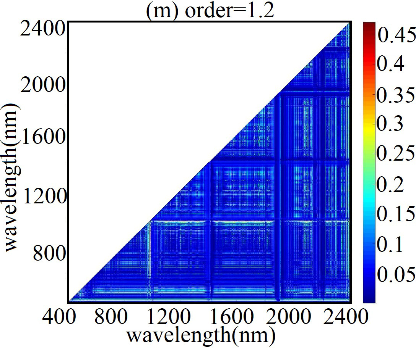

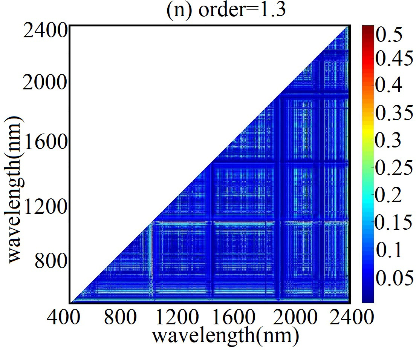

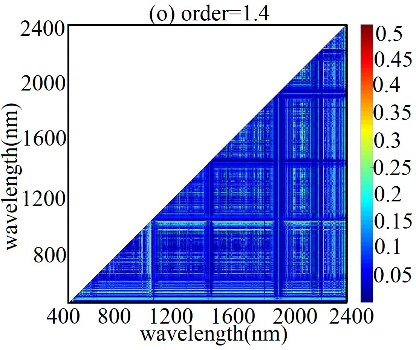

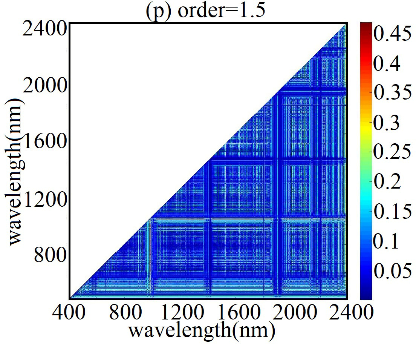


**Fig S5 Correlation between the Brightness index (BI))and Ni content for different orders: 0-order(a), 0.1-order(b), 0.2-order(c), 0.3-order(d), 0.4-order(e), 0.5-order(f), 0.6-order(g), 0.7-order(h), 0.8-order(i), 0.9-order(j), 1.0-order(k), 1.1-order(l), 1.2-order(m), 1.3-order(n), 1.4-order(o), 1.5-order(p), 1.6-order(q), 1.7-order(r), 1.8-order(s), 1.9-order(t), and 2.0-order(u).**
